# Radiotherapy may improve overall survival of patients with T3/T4 transitional cell carcinoma of the renal pelvis or ureter and delay bladder tumour relapse

**DOI:** 10.1186/1471-2407-11-297

**Published:** 2011-07-14

**Authors:** Bing Chen, Zhao-Chong Zeng, Guo-Min Wang, Li Zhang, Zong-Ming Lin, Li-An Sun, Tong-Yu Zhu, Li-Li Wu, Jian-Ying Zhang, Yuan Ji

**Affiliations:** 1 Department of Radiation Oncology of Zhongshan hospital, Fudan University, 136 Yi Xue Yuan Road, Shanghai 200032, China; 2 Department of Urology of Zhongshan hospital, Fudan University, 136 Yi Xue Yuan Road, Shanghai 200032, China; 3 Department of Pathology of Zhongshan hospital, Fudan University, 136 Yi Xue Yuan Road, Shanghai 200032, China

## Abstract

**Background:**

Since transitional cell carcinoma (TCC) of the upper urinary tract is a relatively uncommon malignancy, the role of adjuvant radiotherapy is unknown.

**Methods:**

We treated 133 patients with TCC of the renal pelvis or ureter at our institution between 1998 and 2008. The 67 patients who received external beam radiotherapy (EBRT) following surgery were assigned to the radiation group (RT). The clinical target volume included the renal fossa, the course of the ureter to the entire bladder, and the paracaval and para-aortic lymph nodes, which were at risk of harbouring metastatic disease in 53 patients. The tumour bed or residual tumour was targeted in 14 patients. The median radiation dose administered was 50 Gy. The 66 patients who received intravesical chemotherapy were assigned to the non-radiation group (non-RT).

**Results:**

The overall survival rates for the RT and non-RT groups were not significantly different (p = 0.198). However, there was a significant difference between the survival rates for these groups based on patients with T3/T4 stage cancer. A significant difference was observed in the bladder tumour relapse rate between the irradiated and non-irradiated bladder groups (p = 0.004). Multivariate analysis indicated that improved overall survival was associated with age < 60 years, T1 or T2 stage, absence of synchronous LN metastases, and EBRT. Acute gastrointestinal and bladder reactions were the most common symptoms, but mild non-severe (> grade 3) hematologic symptoms also occurred.

**Conclusion:**

EBRT may improve overall survival for patients with T3/T4 cancer of the renal pelvis or ureter and delay bladder tumour recurrence in all patients.

## Background

Transitional cell carcinoma (TCC) of the renal pelvis and ureter is a relatively uncommon malignancy; it is estimated to account for 7% of all renal neoplasms and 5% of all urothelial malignancies in the United States [1]. Upper urinary tract carcinoma is often a multifocal process, meaning that patients with cancer localised to the upper urinary tract are at greater risk of developing transitional tumours elsewhere. Approximately 20-50% of patients with an upper urinary tract tumour will develop bladder cancer [2-4].

Radical nephroureterectomy is a routine initial therapy for most patients with TCC of the renal pelvis or ureter. Surgery alone provides sufficient loco-regional control for the majority of patients that present with early stage disease; however, the overall 5-year survival rate after surgery ranges from 0-34% for patients with locally advanced TCC of the renal pelvis and ureter. The rate of local failure is reported to be between 30 and 50%, and this is the major cause of mortality in TCC patients [2-5].

The role of adjuvant external beam radiotherapy (EBRT) in the treatment of patients with TCC of the renal pelvis and ureter is unknown. Some studies report that EBRT is not effective; therefore, EBRT is not recommended for use as a prophylactic postoperative therapy [6,7]. In contrast, other studies report that EBRT may improve the treatment outcome when it is concurrently administered with chemotherapy in patients with resected, locally advanced, upper tract urothelial malignancies. The ability of EBRT to improve the treatment outcome for patients with locally advanced renal pelvis or ureter cancer has been assessed [8,9], but the studies included a small patient population and were not detailed enough to draw any conclusions. In this study, we report on a relatively large group of patients with TCC of the renal pelvis and ureter who were treated with postoperative radiotherapy and intravesical chemotherapy. Therefore, this study provides a detailed evaluation of the efficacy of adjuvant radiotherapy in patients with TCC of the renal pelvis and ureter.

## Methods

### Patients and staging evaluation

To evaluate the outcome of external beam radiation therapy (EBRT) in patients with TCC of the renal pelvis or ureter, we analysed data collected prospectively for patients treated at the Department of Urology at Zhongshan Hospital, Fudan University between September 1998 and April 2008. The study was approved by the Fudan University Zhongshan Hospital Ethics Committee. A total of 133 patients were included in this study. The patients had nephroureterectomy without distant metastases, and were pathologically re-staged according to the sixth American Joint Committee on Cancer (AJCC) staging manual [10]. Patients who died within the peri-operation period (6 weeks) or who developed ureteral cancer from bladder cancer or bladder tumour recurrence within 3 months following nephroureterectomy were excluded. Due to conflict among urologists regarding the outcome of adjuvant post-operative EBRT for treating TCC of the renal pelvis or ureter, the decision of whether a patient should receive radiation therapy was based on the physician's preference and the patient's consent. Since this factor could have potentially contributed a significant bias to our study, we compared the demographic and clinical factors of patients who did and did not receive radiation therapy (Table 1).

**Table 1:** Patient characteristics

	n	Non-RT	RT	*p *value
Age (average)		68.5 ± 1.430	66.2 ± 1.208	0.074
< 60 years	31	14(21.2%)	17(25.4%)	0.570
≥60 years	102	52(78.8%)	50(74.6%)	
Sex				
Female	50	28(42.4%)	22(32.8%)	0.254
Male	83	38(57.6%)	45(67.2%)	
Tumor location				
Renal pelvis	42	26(39.4%)	16(23.9%)	0.054
Ureter	91	40(60.6%)	51(76.1%)	
Tumor lesions				
Solitary	117	61(92.4%)	56(83.6%)	0.117
Multiple nodules	16	5(7.6%)	11(16.4%)	
Pathologic T stage				
T1	21	12(18.2%)	9(13.4%)	0.083
T2	60	30(45.5%)	30(44.8%)	
T3	33	11(16.7%)	22(32.8%)	
T4	19	13(19.7%)	6(9.0%)	
Synchronous LN				
-	124	61(92.4%)	63(94.0%)	0.712
+	9	5(7.6%)	4(6.0%)	
Resection modality				
Local resection	22	17(25.8%)	5(7.5%)	0.004
Nephroureterectomy	111	49(74.2%)	62(92.5%)	
Residual Disease				
R0	108	53(80.3%)	55(82.1%)	0.816
R1	11	5(7.6%)	6(9.0%)	
R2	14	8(12.1%)	6(9.0%)	
Histological grade				
I	6	5(7.6%)	1(1.5%)	0.195
II	73	38(57.6%)	35(52.2%)	
III	51	21(31.8%)	30(44.8%)	
IV	3	2(3.0%)	1(1.5%)	

RT: Radiation Therapy, LN: Lymph Node

### Therapies

Surgical resection: Before surgical resection, all patients were required to undergo computed tomography (CT) and/or magnetic resonance imaging (MRI) of the abdomen and pelvis, and an intravenous excretory urogram or retrograde pyelogram. Of the 133 patients in the study, 108 were treated with the standard approach of radical nephroureterectomy, including removal of the contents of Gerota's fascia with the ipsilateral ureter and the cuff of the bladder at its distal extent. Open nephron-sparing surgery for upper-tract TCC was used in patients with a large renal pelvic tumour in a single kidney. A partial ureterectomy or distal ureterectomy with reimplantation is a reasonable treatment alternative for patients who underwent palliative resection (R2, macroscopic residual) or had poor kidney function. Twenty-five patients underwent local resection surgery, including nine patients with gross lymph node metastases or accompanying cancers, for which palliative resection was subsequently performed. There were five patients over 80 years of age, four patients with poor kidney function or an absent contralateral kidney, two patients mistakenly diagnosed with renal cell cancer and underwent radical nephrectomy, two patients mistakenly diagnosed with urolithiasis but were subsequently found to have TCC, and two patients with tumour invasion to psoas.

Intravesical chemotherapy: All patients underwent a 8-week induction regimen followed by a once-monthly maintenance regimen for 12 months. The induction regimen consisted of intravesical administration of mitomycin C (20 mg) or epirubicin (50 mg) dissolved in 50 ml saline, which was retained for 60 minutes and drained once a week for 8 weeks. This procedure was followed by the maintenance regimen, which used the same dose of mitomycin or epirubicin once a month for 12 months.

**Figure 1:**
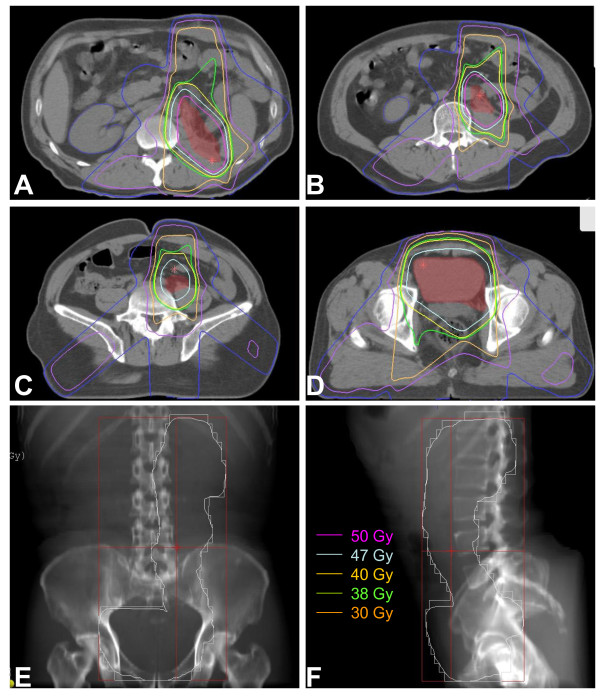
**Dose distribution of a patient with renal pelvis cancer and beam arrangements of 0°, 129°, and 229° gantry: (A) renal fossa; (B, C) course of ureter; and (D) bladder**. Digitally reconstructed radiograph for views of (E) 0° gantry and (F) 90° gantry. Internal pink and yellow lines represent the CTV50 and CTV40 respectively.

Post-operative radiotherapy: Each patient provided written or oral informed consent regarding their treatment course. The median and average interval between nephroureterectomy or ureterectomy and EBRT was 23 and 26 days (range, 12-76 days, standard error, 1.55 days) respectively. Patients received limited-field EBRT using a linear accelerator with 15-MV photons. Immobilization devices for patients with intra-abdominal tumours usually included the thorax and pelvis. Radiation doses of 46 to 50 Gy at 2 Gy per day were routinely used to treat subclinical disease. For R1 (microscopic positive margins) or R2 (macroscopic residual margins) resections, a boost of 6 to 10 Gy at 2 Gy per fraction was considered. The median dose of EBRT administered to patients in this study was 50 Gy (range, 36-60 Gy). Multiple-field arrangements, including oblique and lateral fields with field reductions, were important to minimise the toxicity to surrounding normal tissues. The clinical target volume covered the ipsilateral renal fossa and the course of the ureter, the whole bladder, and the paracaval and para-aortic lymph nodes, which were at risk of harbouring metastatic disease in 53 patients. In addition, the tumour bed and regional draining lymph nodes were targeted in 14 patients, including six patients who underwent R1 or R2 resection and eight patients by request. A coverage area of 90.0% of the planned treatment volume as defined by the 95% isodose line was required. Figure 1 shows the designated irradiation fields, including the renal fossa, ureter, whole bladder, and the at-risk lymph nodes.

### Assessment of toxicity

Patients were monitored weekly by radiation oncologists for symptoms of toxicity resulting from their radiation therapy. Radiation toxicity was evaluated according to the guidelines established by the Radiation Therapy Oncology Group (RTOG; version 2.0) [11].

### Follow-up

Patients were advised of the need for an initial follow-up examination at the sixth week after completion of EBRT. Patients were monitored every 3 to 6 months thereafter. Follow-up information was obtained primarily through telephone interview and follow-up was stopped on March 15, 2009. The overall survival time was defined as the interval between the date of surgery and either the date of death or the date of the last follow-up (censored).

### Statistical analysis

The primary endpoint measures of the study were safety, loco-regional failure or the occurrence of distant metastasis, and overall survival. These were defined as the time from the date of nephroureterectomy. Data were censored at the date of the last visit or the date of death. The characteristics of the patients, loco-regional failure or metastasis rates, and overall survival rates were compared between patients in the RT and non-RT groups. The chi-square test was used for comparison between groups. Survival curves were calculated by the Kaplan-Meier method and compared using the log-rank test. The Cox proportional hazards model was used to determine the independent factors affecting endpoints, based on the variables selected by the univariate analysis. Statistical analyses were performed by SPSS 13.0 for Windows (SPSS Inc., Chicago, IL). P values < 0.05 were considered to be statistically significant.

## Results

Among the 133 total subjects, 53 died during follow-up, four were lost during follow-up, and the remaining 76 were censored at the end of the follow-up period on 3/15/2009. The interval between surgery and initiation of RT was 26.4 days on average with a 95% confidence interval of ± 3.14 days. The median follow-up time was 26.6 months. The age of the patients ranged from 34 to 91 years, and the mean age was 67.8 years. A similar proportion of patients with similar clinical symptoms were included in the radiation (RT) and non-radiation (non-RT) groups (Table 1).

We scheduled the full postoperative adjuvant radiation dose as 46-50 Gy, but this dosage was subject to change based on unpredicted situations that arose during the course of EBRT and indicated the need for a reduced dose, such as adverse side effects and the emergence of distant metastases. The dosage was increased to between 54 and 60 Gy for patients who underwent R1 or R2 resection (12 patients). Three patients presented with distant metastases during radiation, and four patients developed ≥ grade 2 anorexia; the radiation dose was reduced to < 46 Gy in these seven cases.

### Survival analyses and prognostic factors

**Figure 2:**
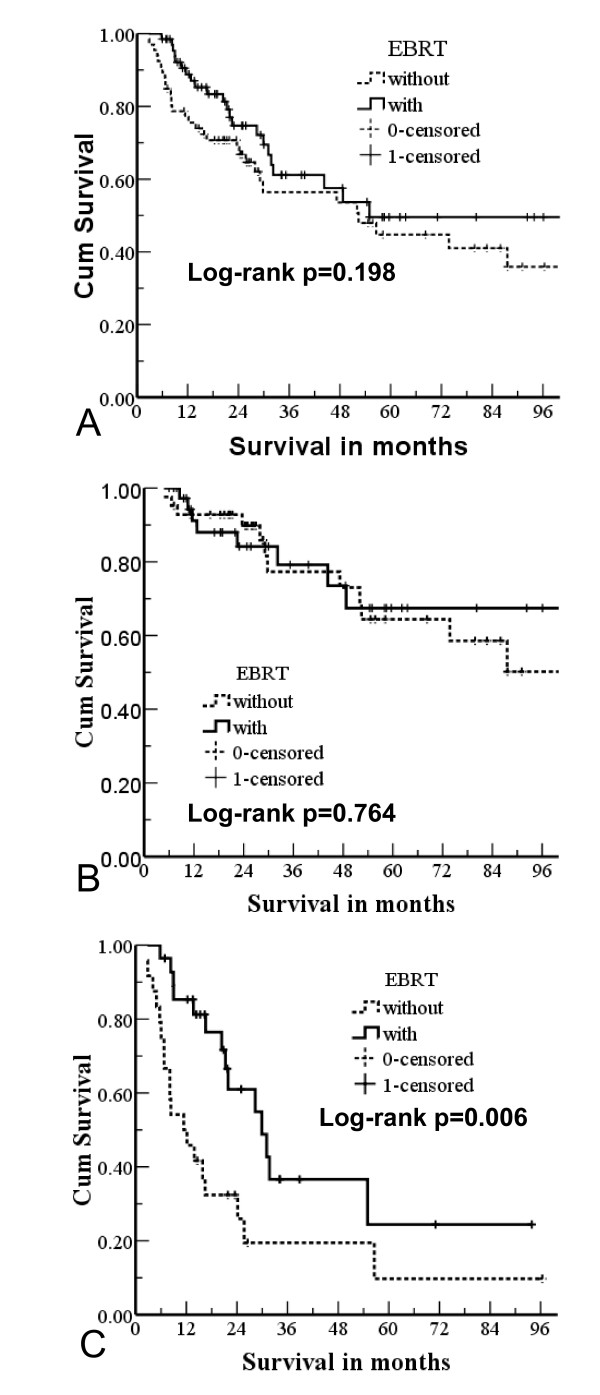
**Survival curves of external beam radiotherapy (EBRT) and non-EBRT groups based on (A) all tumour stages; (B) T1/T2 stage; and (C) T3/T4 stage**.

The median survival period was 55.0 months (range, 2.8-118.6). The overall 1-, 3-, and 5-year survival rates were 82.2%, 58.8%, and 47.1%, respectively. Of the 133 patients, 67 were in the RT group and 66 were in the non-RT group. The overall survival rates for these two groups at 1, 3, and 5 years were 98.8% vs. 75.5%, 61.1% vs. 53.6%, and 49.6% vs. 44.7%, respectively. The median survival periods were 55.0 months vs. 52.4 months, respectively. There was no significant difference between the overall survival for the RT and non-RT groups (p = 0.198; Figure 2A).

When we subdivided the groups based on the tumour stage of the patients, we found a significant difference between the RT and non-RT groups for patients who had a T3 or T4 tumour stage. The median survival times of these two groups were 29.9 months vs. 11.4 months (p = 0.006) for the RT and non-RT groups, respectively (Figure 2B, C).

Univariate analysis indicated that an increased overall survival rate was significantly associated with age < 60 years, a T1 or T2 tumour stage, no synchronous LN metastases, nephroureterectomy, a R0 resection, and a lower histological grade (Table 2).

**Table 2:** Univariate and multivariate analysis of baseline predictors of survival in 133 patients with transitional cell carcinoma of the renal pelvis and/or ureter

Variables	n	Survival status		*p *value		Correlation with histology	
		
		3-y	5-y	Univariate	Multivariate	coefficient	*p*
Age						0.130	0.136
< 60 years	31	66.8	66.8	0.029	0.015		
≥ 60 years	102	52.5	37.4				
Sex						0.136	0.119
Female	50	59.3	48.4	0.402	0.153		
Male	83	53.6	35.8				
Tumor location						-0.064	0.463
Renal pelvis	42	59.3	59.3	0.912	0.346		
Ureter	91	57.0	38.9				
Tumor lesions						0.024	0.785
Solitary	117	55.0	42.9	0.612	0.340		
Multiple nodules	16	52.9	26.4				
pT stage						0.404	< 0.001
T1 or T2	81	76.1	61.6	< 0.001	0.001		
T3 or T4	52	22.2	16.7				
Synchronous LN						0.143	0.100
-	124	61.7	47.8	< 0.001	0.002		
+	9	0	0				
Resection modalities						-0.250	0.004
Local resection	22	33.7	33.7	0.024	0.178		
Nephroureterectomy	111	63.4	50.0				
Residual Disease						0.347	< 0.001
R0	108	65.9	52.2	< 0.001	0.077		
R1 or R2	25	10.7	10.7				
Histological grade							
Grade I or II	79	71.1	56.0	< 0.001	0.465		
Grade III or IV	54	33.2	28.4				
EBRT						0.116	0.182
No	66	53.6	41.0	0.198	0.024		
Yes	67	57.5	49.6				

LN: Lymph Node; EBRT: External Beam Radiation Therapy

Using multivariate analysis, we identified several favourable predictors associated with an improved overall survival, which included age < 60 years, a T1 or T2 tumour stage, absence of synchronous LN metastases, and EBRT. The histological grade factor was determined to be significantly different using univariate analysis but not significantly different based on multivariate analysis. This result is due to multicollinearity, which arises because many of the evaluated risk factors correlate with histological grade. In this study, the histological grade correlated with the tumour stage, resection models, and presence of residual disease (Table 2).

### Failure patterns

**Figure 3:**
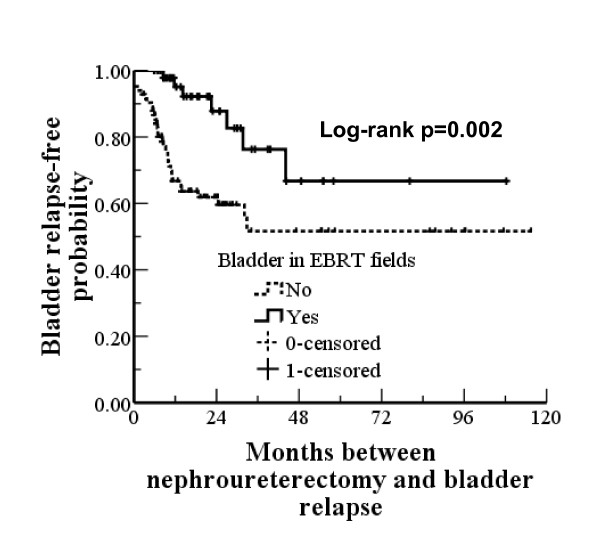
**Bladder tumour relapse-free probability for patients treated with or without bladder irradiation**.

In this study, 38 (28.6%) patients experienced bladder tumour relapse. Table 3 shows the univariate and multivariate analysis for patients with and without bladder tumour relapse. The favourable predictors were associated renal pelvic TCC and irradiation of bladder. Bladder tumour relapse rates in the non-RT and RT groups were 34.8% (23/66) vs. 22.4% (15/67), respectively; however, this difference was not statistically significant (p = 0.112). Of the 67 patients who received EBRT, 14 patients did not undergo bladder irradiation; their radiation treatment focused only on the tumour bed due to either R1 or R2 resection or the patient's request. When we sub-divided the 133 patients based on bladder irradiation, a significant difference was observed in the rate of bladder tumour relapse between the two groups [38.7% (31/80) without irradiation vs. 13.2% with irradiation (7/53) (chi-square test, p = 0.001), respectively]. Figure 3 provides the bladder tumour relapse-free survival curves. The curves stabilised to approximately 50.0% after 32.8 months in patients who had not received bladder irradiation and to approximately 69.1% after 44.1 months in patients who had received bladder irradiation. There was a significant difference between the two groups (p = 0.002).

**Table 3:** Univariate and multivariate analysis of predictors of bladder tumor relapse in 133 patients with TCC of the renal pelvis or ureter

Variables	n	Bladder tumor relapse		X^2^	*p *values	
		Absent (n = 95)	Present (n = 38)		univariate	multivariate
Age						
< 60 years	31	23(74.2%)	8(25.8%)	0.697	0.573	0.562
≥ 60 years	102	72(70.6%)	30(29.4%)			
Sex						
Female	50	33(66.0%)	17(34.0%)	0.282	0.277	0.588
Male	83	62(74.7%)	21(25.3%)			
Tumor location						
Renal pelvis	42	36(85.7%)	6(14.3%)	0.013	0.048	0.015
Ureter	91	59(64.8%)	32(35.2%)			
Tumor lesions						
Solitary	117	83(70.9%)	34(29.1%)	0.736	0.628	0.352
Multiple nodules	16	12(75.0%)	4(25.0%)			
pT classification						
T1 or T2	81	59(72.8%)	22(27.2%)	0.653	0.255	0.256
T3 or T4	52	36(69.2%)	16(30.8%)			
Synchronous LN						
-	124	88(71.0%)	36(29.0%)	0.662	0.687	0.759
+	9	7(77.8%)	2(22.2%)			
Resection models						
Local resection	22	17(77.3%)	5(22.7%)	0.507	0.853	0.543
Radical resection	111	78(70.3%)	33(29.7%)			
Residual Disease						
R0	108	78(72.2%)	30(27.8%)	0.674	0.148	0.167
R1 or R2	25	17(68.0%)	8(32.0%)			
Histological grade						
Grade I or II	79	55(69.6%)	24(30.4%)	0.577	0.926	0.816
Grade III or IV	54	40(74.1%)	14(25.9%)			
EBRT						
Non-EBRT	66	43(65.2%)	23(34.8%)	0.112	0.060	0.514
EBRT w/o bladder	67	52(77.6%)	15(22.4%)			
RT including bladder						
No	80	49(61.3%)	31(38.7%)	0.001	0.002	< 0.001
Yes	53	46(86.8%)	7(13.2%)			

No lymph node metastasis was found in pT1 or T2 patients, so patients with T3 or T4 are equal to those with AJCC stage III or IV in this study. We did not use AJCC Stage III or IV in this table, because we excluded patients with distant metastases from our study, who are also regarded as stage IV.

TCC: Transitional Cell arcinoma; LN: Lymph Node; EBRT: External Beam Radiation Therapy

Table 4 provides the failure patterns for the two groups. EBRT significantly reduced the rate of loco-regional relapse, including anatomic/tumour bed recurrence and lymph node metastases, in patients who underwent nephroureterectomy.

**Table 4:** Comparison of the failure patterns between the two groups*

	Non-Radiation (n = 66)	Radiation (n = 67)	*p *values
Anatomic/tumor bed recurrence	4(6.9%)^1^	0(0%)^2^	0.044
LN metastases	15 (24.6%)^3^	6(9.5%)^4^	0.025
Bladder recurrence	23(34.8%)	15(22.4%)	0.112
Distant metastases	15(22.7%)	16(23.9%)	0.875

* There were 94 sites of relapse in 64 patients, including 36 and 28 patients, for the Non-RT and RT groups, respectively. LN: Lymph Node

1. Eight R1 or R2 patients with primary lesions were excluded.

2. Eight RI or R2 patients with primary lesions were excluded.

3. Five patients with synchronous LN metastases were excluded.

4. Four patients with synchronous LN metastases were excluded.

### Adverse effects

Acute gastrointestinal reactions occurred in most patients with RTOG grade 1 and were uncommon in patients with grade 2. The most common side effect was bladder spasms with mild symptoms not requiring intervention. There were no cases of gastrointestinal bleeding or perforation, and there were no severe cases (> grade 3) of haematologic symptoms (Table 5).

**Table 5:** Side Effects of Radiation Therapy in 67 Patients

Side Effect	RTOG grade (*n*)			
	
	1	2	3	4
Gastrointestinal				
Anorexia	34(50.7%)	2(3.0%)	2(3.0%)	0
Vomiting	19(28.4%)	3(4.5%)	0	0
Diarrhea	18(26.9%)	4(6.0%)	0	0
Bladder				
Spasms	35(52.2%)	4(6.0%)	2(3.0%)	0
Bone marrow				
WBC	23(34.3%)	10(14.9%)	2(3.0%)	0
Platelets	17(25.4%)	8(11.9%)	5(7.5%)	0
Hemoglobin	12(17.9%)	6(9.0%)	2(3.0%)	0

RTOG: Radiation Therapeutic Oncology Group; WBC: White Blood Counts.

### Causes of death

Table 6 lists the causes of patient death. The most common cause of death was distant metastases; however, not this was not the cause of death in all patients. The causes of death were compared between patients in the RT and non-RT groups, and no significant differences were identified (p = 0.461).

**Table 6:** Causes of Death in 53 Patients with TCC of the Renal Pelvis or Ureter

Causes	Non-radiation group (n = 30)	Radiation group (n = 23)	*p *value
Distant metastases	18(60.0%)	17(73.8%)	0.461
Lung	13	11	
Liver	3	3	
Bone	2	3	
Abdominal LN metastases	8(26.7%)	3(13.1%)	
Death unrelated to cancer	4(13.3%)	3(13.1%)	

TCC: Transitional Cell Carcinoma; LN: Lymph Node.

## Discussion

Although nephroureterectoumy is a curative therapy for the majority of patients who present with tumours limited to the ureteral muscularis, the rate of local-regional recurrence may be as high as 45% (57/126), as reported in a median 9-month follow-up study in patients with locally advanced disease [6]. In addition, persistent loco-regional disease may play an important role in the subsequent development of distant metastases. Therefore, it is important to address the role of adjuvant radiation therapy, in addition to surgery, for the local and regional control of the disease, as well as to improve the overall treatment outcome. TCC of the renal pelvis and ureter is an unusual disease with insufficient clinical data; therefore, the role of EBRT in treating this disease is unclear. Czito et al. [9] retrospectively reviewed the records of 31 patients with locally advanced TCC of the renal pelvis and ureter who underwent surgery followed by adjuvant EBRT with or without concurrent chemotherapy [methotrexate, cisplatin and vinblastine (MCV)]. The 5-year overall and disease-free survival rates were improved in patients with locally advanced upper tract urothelial malignancies who underwent resection surgery and adjuvant concurrent chemoradiotherapy. This regimen should be considered in patients with T3/4 and/or node positive upper tract TCC. Patients with advanced disease treated with surgery alone had a shorter disease-free survival (23.3 months) than those treated with combination chemoradiotherapy (45.2 months). In our study, we also found that radiotherapy significantly reduced the rate of tumour recurrence, irrespective of the pT stage or the presence of synchronous LN. However, EBRT seemed to confer an overall survival advantage only to patients with T3/T4 stage or synchronous LN.

In this study, univariate analysis indicated that an increased overall survival was significantly associated with age < 60 years, a T1 or T2 tumour stage, no synchronous LN metastases, nephroureterectomy, R0 resection and a lower histological grade. These prognostic factors are similar to those reported by Ozsahin et al. [6], Tan et al. [3], and Raman et al. [12]. Recently, Raman et al. reported a retrospective review of data collected from 10 global institutions, which included 1,249 patients with TCC of the renal pelvis and ureter. This study determined that advanced T stage, higher histological grade, and lymph node metastases were associated with a poorer survival outcome [12].

Both our univariate and multivariate analyses suggested that radiation was an effective and tolerable treatment for patients with T3 or T4 pelvis or ureteral TCC. However, there are three fundamental questions that must be answered in order to develop a comprehensive plan for performing radiotherapy in these patients:

1) What is the appropriate volume of tissue that we must irradiate to achieve the desired curative or palliative goal? The volume of tissue exposed to radiation in order to encompass the gross nodal metastases and the residual tumour, and whether to include adjacent areas of potential microscopic disease, have been controversial issues. In this study, the clinical target volume included the renal fossa, the course of the ureter to the bladder, and the paracaval and para-aortic lymph nodes, which were at risk of harbouring metastatic disease. For T1 or T2-stage patients, radiotherapy did not improve overall survival, but did delay bladder tumour relapse.

Bladder tumour recurrence following nephroureterectomy for TCC of the upper urinary tract was observed in 30-40% of patients [2,3]. Most bladder tumour recurrences develop within 2 years following surgical resection. Cozad et al. reported that radiotherapy could significantly reduce local recurrence. There was a borderline significant increase in the overall survival (p = 0.07) for 77 patients who all had T stage renal pelvis and ureteral cancer [13]. Multivariate analysis determined that the ureteric tumour location was an independent predictor (p = 0.02) for bladder tumour relapse, with rates of 20% (25/123) for patients with renal pelvis cancer and 40% (19/47) for patients with ureteral cancer. Our results showed that the bladder tumour relapse rates were 14.3% (6/42) for renal pelvis cancer and 35.2% (32/91) for ureteric cancer (chi-square test, p = 0.013). Based on these observations, we suggest that the clinical target volume should only include the bladder for elective radiotherapy in the case of T1 or T2 patients, especially in patients with ureteric cancer, as this regimen will effectively delay bladder tumour relapse.

2) What is the planned treatment dose? Selection of the radiation dose is a complex issue for abdominal cancer, because it requires the radiation oncologist to weigh the use of a sufficiently high radiation dose against an unacceptably high risk of side effects. Radiation complications consistently increase as the radiation dose increases. In general, the post-operation adjuvant radiation dose should be between 45 and 50 Gy, as this was established to be a safe dosage for the small bowel and colon. Usually, the length of the field from cephalic to caudal is >30 cm; however, no grade toxicity was observed in this study. Chauffert et al. reported no gastrointestinal bleeding in 119 patients with advanced pancreatic cancer who received 60 Gy radiation with conventional fractions [14]. Therefore, a radiation dose between 45 and 50 Gy was acceptable to use in this study.

3) What are the chemotherapeutic regimens? Adjuvant chemotherapy with paclitaxel and carboplatin [15] or M-VAC [16] is feasible, may reduce the risk of distant metastases, and may prevent the recurrence of bladder tumour in patients with high-risk upper urinary tract carcinoma. There are a few controlled clinical trials using adjuvant or radical radiotherapy with or without chemotherapy in patients with renal pelvic and ureteric cancer; therefore, whether radiotherapy improves the treatment outcome is inconclusive. A study by Czito et al. reported that cisplatin-based chemotherapy concurrently delivered with radiation significantly improved the treatment outcome. Chemotherapy may further improve the treatment outcome if it is concurrently delivered with high-dose radiation. Unfortunately, we did not adopt adjuvant chemotherapy in this study, partly due to the lack of evidence supporting adjuvant chemotherapy at the time of our protocol design. However, all patients in this study received postoperative instillation of MMC or epidorubicin as a regional chemotherapy, and this regimen successfully reduced the recurrence of bladder tumours after surgery for upper urinary tract tumours [17]. Currently, there is not enough evidence to support replacement of systemic chemotherapy with intravesical chemotherapy.

Limitations of our study included the non-randomised design and the use of several different radiation techniques, which may have been affected by selection bias, and the fact that no systemic chemotherapy regimens were included. Although our sample size was relatively large, these issues should be addressed, preferably in a prospective study using a randomised design. However, due to the rarity of this malignancy, development of the optimal treatment strategy for patients with TCC of the renal pelvis and ureter may be impossible without collaborative efforts among multiple cancer treatment institutions.

## Conclusions

The use of adjuvant EBRT was efficacious in the treatment of TCC of the renal pelvis or ureter, as it reduced the local recurrence for all patients and improved the overall survival for those patients with T3/T4 classification. Bladder tumour recurrence was the main cause of local recurrence, so the clinical target volume should include the whole bladder for all tumour stages. Radiotherapy and chemotherapy used in an adjuvant setting is a strategy that warrants optimization and evaluation in a prospective, randomised clinical trial setting to determine whether it can further improve treatment outcome.

Impact of tumor location on prognosis for patients with upper tract urothelial carcinoma managed by radical nephroureterectomy.

## Competing interests

The authors declare that they have no competing interests.

## Authors' contributions

Z-CZ and BC conceived and designed the study; Z-CZ provided financial support, Z-CZ and T-YZ provided administrative support; Z-CZ, BC, G-MW, LZ, Z-ML, L-AS, and YJ provided study materials; BC, Z-CZ, L-LW collected and assembled data; BC, Z-CZ, L-LW, and J-YZ analysed the data; and BC and Z-CZ wrote the manuscript. All authors read and approved the final manuscript.

## Pre-publication history

The pre-publication history for this paper can be accessed here:

http://www.biomedcentral.com/1471-2407/11/297/prepub
